# Chitosan/Alginate Nanoparticles for the Enhanced Oral Antithrombotic Activity of Clam Heparinoid from the Clam *Coelomactra antiquata*

**DOI:** 10.3390/md20020136

**Published:** 2022-02-12

**Authors:** Guan-Lan Chen, Hong-Ying Cai, Jian-Ping Chen, Rui Li, Sai-Yi Zhong, Xue-Jing Jia, Xiao-Fei Liu, Bing-Bing Song

**Affiliations:** 1 Guangdong Provincial Key Laboratory of Aquatic Product Processing and Safety, Guangdong Province Engineering Laboratory for Marine Biological Products, Guangdong Provincial Engineering Technology Research Center of Seafood, Guangdong Provincial Science and Technology Innovation Center for Subtropical Fruit and Vegetable Processing, College of Food Science and Technology, Guangdong Ocean University, Zhanjiang 524088, China; cgl202112@163.com (G.-L.C.); 13414866246@163.com (H.-Y.C.); cjp516555989@gdou.edu.cn (J.-P.C.); liruihn@163.com (R.L.); jiaxj@gdou.edu.cn (X.-J.J.); liuxf169@126.com (X.-F.L.); 15891793858@163.com (B.-B.S.); 2 Guangdong Province Key Laboratory of Aquatic Products Processing and Safety, Guangdong Province Engineering Laboratory for Marine Biological Products, School of Food Science and Technology, Guangdong Ocean University, Zhanjiang 524088, China; 3 Shenzhen Institute, Guangdong Ocean University, Shenzhen 518108, China; 4 Collaborative Innovation Center of Seafood Deep Processing, Dalian Polytechnic University, Dalian 116034, China

**Keywords:** clam heparinoid, oral administration, antithrombotic, chitosan/alginate nanoparticles

## Abstract

Chitosan/alginate nanoparticles (DG1-NPs and DG1/Cur-NPs) aiming to enhance the oral antithrombotic activity of clam heparinoid DG1 were prepared by ionotropic pre-gelation. The influence of parameters, such as the concentration of sodium alginate (SA), chitosan (CTS), CaCl_2_, clam heparinoid DG1, and curcumin (Cur), on the characteristics of the nanoparticles, were investigated. Results indicate that chitosan and alginate can be used as polymer matrices to encapsulate DG1, and nanoparticle characteristics depend on the preparation parameters. Nano-particles should be prepared using 0.6 mg/mL SA, 0.33 mg/mL CaCl_2_, 0.6 mg/mL CTS, 7.2 mg/mL DG1, and 0.24 mg/mL Cur under vigorous stirring to produce DG1-NPS and DG1/Cur-NPS with small size, high encapsulation efficiency, high loading capacity, and negative zeta potential from approximately −20 to 30 mV. Data from scanning electron microscopy, Fourier-transform infrared spectrometry, and differential scanning calorimetry analyses showed no chemical reaction between DG1, Cur, and the polymers; only physical mixing. Moreover, the drug was loaded in the amorphous phase within the nanoparticle matrix. In the acute pulmonary embolism murine model, DG1-NPs enhanced the oral antithrombotic activity of DG1, but DG1/Cur-NPs did not exhibit higher antithrombotic activity than DG1-NPs. Therefore, the chitosan/alginate nanoparticles enhanced the oral antithrombotic activity of DG1, but curcumin did not further enhance this effect.

## 1. Introduction

Venous thromboembolism (VTE), including deep vein thrombosis and pulmonary thromboembolism, represents the third leading vascular disease after acute myocardial infarction and stroke [1,2]. Heparin (HP) is a widely used anticoagulant owing to its excellent effects for preventing and treating VTE [3]. HP is administered by the parenteral route, which is inconvenient and leads to lower patient compliance compared to other routes. However, degradation by gastric acid, heparanase in the liver, and poor absorption and utilisation by the intestinal epithelium and lymph caused by the considerable molecular weight and negative charge of HP lead to a low oral absorption [4]. Although oral anticoagulants such as vitamin K, dabigatran, rivaroxaban, apixaban, and edoxaban are available on the market, HP is still the most effective anticoagulant [5]. Therefore, highly orally active oral formulations of HP are urgently needed to be developed.

Heparinoid, also known as “mucopolysaccharide polysulfated,” refers to heparin, acetyl heparan sulfate (HS), heparin-like molecules of plant and animal origin, and sulfated polysaccharides, among other sulfated polysaccharides that have similar structure and properties to heparin [6]. The current production of commercial heparins suffers from insufficient resources, biological contamination, and susceptibility to bleeding side effects. The development of marine origin heparinoids is one of the strategies to remedy these problems [7]. The heparinoid G15 from the clam *Coelomactra antiquata* is a homogeneous glycosaminoglycan with a potent anticoagulant and fibrinolytic activity, and it is mainly composed of →4)-α-IdoA2S-(1→4)-α-GlcNS3S6S (or GlcNS6S)-(1→4)-β-GlcA-(1→4)-α-GlcNS6S (or GlcNAC)-(1→ [8]. G2 is prepared by using the extraction of G15 as a reference, and DG1 is a low molecular weight fragment of the vitamin-catalysed free-radical depolymerisation of G2. In a previous study, in a mice blacktail model, we found that gavage of equal doses of DG1, G2, and heparin sodium had a comparable ability to reduce the blacktail ratio in mice [9].

Curcumin (Cur) used as a spice, food coloring, and traditional herbal medicine, is a natural polyphenolic component of *Curcuma longa*, has been widely used in complementary and alternative medicine because it is non-toxic, safe, and has antioxidant, lipid-regulating, anti-platelet aggregation, antithrombotic, etc. properties [10,11,12]. Considering the low availability of heparin from clam *Coelomactra antiquate* [8], in this study, curcumin and clam heparinoid DG1 were used as oral antithrombotic agents to develop oral formulations with high oral antithrombotic activity to provide a reference for the application of heparinoids of marine animal origin. 

Polymer- and lipid-based nanocarriers, such as polymeric micelles, polymeric nanoparticles, lipid nanocapsules, microemulsions, and solid lipid nanoparticles, have been investigated to improve the HP oral absorption [13,14,15,16,17]. Chitosan and alginate biopolymers have received significant attention in this context because of their non-toxic, biocompatible, and biodegradable properties [18,19]. Sodium alginate (SA) is a pH-sensitive material with protonation and dissolution properties under acidic and alkaline conditions, respectively, which prevents drug degradation by gastric acid and provides a targeted release in the intestinal tract. Chitosan (CTS), which is deacetylated from chitin, can enhance the permeation effect and control the release of drugs in the intestinal tract [20,21]. Yin et al. reported the production of sodium alginate/chitosan composite nanoparticles loaded with chondroitin sulfate, in which most CS passed through the stomach and was released in the intestinal tract [22]. The study by Pitchaya et al. confirmed that hydroxyethyl chitosan inhibited the burst release of paracetamol from hydroxyethyl chitosan/SA hydrogels in simulated intestinal fluid [23]. According to Li et al., chitosan-alginate nanoparticles presented a pH-responsive release of nifedipine [24]. Similar results were obtained by Thai et al. [25]. Yan et al. reported that oral fucosylated chondroitin sulfate (FCS) oligomer-gastro resistant microcapsules prepared using a chitosan-coated alginate system presented more robust anticoagulant and antithrombotic effects and weaker bleeding side effects than FCS oligomers [26]. Apart from that, sodium alginate/chitosan nanoparticles have been used to deliver heparin [13] and Cur [27] to improve their bioavailability.

This study used clam heparinoid DG1 and Cur as an oral antithrombotic agent. To further improve the efficacy of DG1, chitosan/alginate nanoparticles DG1-NPs were prepared by ionotropic pre-gelation. Cur was encapsulated in DG1/Cur-NPS nanoparticles to investigate whether Cur and DG1 have synergistic antithrombotic effects. Furthermore, the effects of the concentration of components on the size, zeta potential, encapsulation efficiency, and loading capacity of DG1-NPS and DG1/Cur-NPS were systematically investigated. Fourier-transform infrared spectrometry (FTIR) and differential scanning calorimetry (DSC) were used to analyze the interaction and compatibility of DG1, Cur, and other components. Scanning electron microscopy (SEM) was used to observe the surface morphology of the nanoparticles. The effectiveness of the nanoparticles was evaluated in vivo by establishing an acute pulmonary embolism model of mice.

## 2. Results

### 2.1. Physicochemical Properties of DG1

As shown in Table 1, sulfate radicals and uronic acid contents in DG1 were 24.74% and 22.38%, respectively. The particle size and zeta potential of DG1 were 183.9 nm and −25.1 mV. Owing to its high sulfate radicals and uronic acid contents, HP is the biomolecule with the highest negative charge density [28]. Therefore, DG1 is a water-soluble anionic polymer with a high content of uronic acid and sulfate radicals. The maximum initial degradation temperature of DG1 was 246 °C, which indicated that DG1 could withstand the general heating process of the nanoparticle preparation process.

### 2.2. Optimisation of Clam Heparinoid Nanoparticles

#### 2.2.1. Effect of SA Concentration on NPs

In this study, the effect of SA concentration on the size and potential of the nanoparticles was investigated, considering a fixed mass concentration ratio of sodium alginate to calcium chloride to chitosan of 1:0.22:0.15. 

As shown in Table 2, the particle size and polydispersity index (PDI) of NPs gradually increased with SA concentration. In comparison, there was no change in the zeta potential of NPs, which was attributed to a fixed mass concentration ratio. In particular, higher SA concentrations improve their chance of interaction with CTS and Ca2^+^. The zeta potential of NPs is related to the difference between the negative charge carried by SA and the positive charge of calcium chloride and chitosan. The size, PDI, and absolute zeta potential of ideal nanoparticles with high absorption rate and stable condition should be <1000 nm, <1, and −20–30 mV, respectively [29]. SA concentration at 0.6 mg/mL was considered optimal because it yielded an appropriate zeta potential range (−26.4 ± 1.1 mV) nanoparticles with a smaller particles size (236.5 ± 6.6 nm) (Table 2).

#### 2.2.2. Effect of CaCl_2_ Concentration on NPs

The effect of CaCl_2_ on NPs is shown in Table 3. NPs size decrease with CaCl_2_ concentration at CaCl_2_ concentrations below 0.33 mg/mL, and increases with CaCl_2_ concentration at CaCl_2_ concentrations above 0.33 mg/mL. In addition, the zeta potential of NPs increases with Ca^2+^ concentration. These phenomena can be attributed to a small amount of Ca^2+^ that can be bonded to the G fragment of SA and chelated by CS. However, excessive Ca^2+^ causes an increase in nanoparticle zeta potential, causing nanoparticles to repel each other. Therefore, a CaCl_2_ concentration of 0.33 mg/mL was selected to prepare the NPs with minimum size (171.9 ± 1.1 nm) and appropriate zeta potential range (−27.9 ± 1.9 mV).

#### 2.2.3. Effect of CTS Concentration on NPs

As shown in Table 4, NPs size increase with CTS concentration at CTS concentrations below 0.45 mg/mL, and decreases with CTS concentration at CTS concentrations above 0.45 mg/mL. NP zeta potential increased, and the PDI decreased as the CTS concentration increased. Moreover, a CTS increase above 0.6 mg/mL led to the production of aggregated particles and an opaque suspension. This might have occurred owing to CTS forming a compact membrane on the surface of nanoparticles and having a neutralising effect on the negative charge of the nanoparticles. These results indicated that a CTS concentration of 0.6 mg/mL was optimal to prepare NPs with appropriate zeta potential, minimum PDI, and smaller size.

### 2.3. Effect of DG1 Concentration

As shown in Table 5, when the DG1 concentration is below 9.6 mg/mL, the encapsulation efficiency (EE) and the loading capacity (LC) increase with DG1 concentration. The nanoparticle EE and LC of DG1 reached 98.09% and 18.01 U/mg at a concentration of 9.6 mg/mL. When the DG1 concentration increased to 12 mg/mL, the nanoparticle LC increased to 18.89 U/mg, whereas the EE decreased to 90.09%. When the concentration of DG1 was increased to 12 mg/mL, the LC of the nanoparticles increased to 18.89 U/mg, while the EE decreased to 90.09% with the occurrence of agglomeration. Moreover, when the DG1 concentration was below 7.2 mg/mL, the particle size and PDI significantly increased, and zeta potential decreased as the DG1 concentration increased. When the DG1 concentration was 7.2 mg/mL, the nanoparticle size was 252.4 nm. As the DG1 concentration increased to 9.6 mg/mL, the nanoparticle size increased to 366.6 nm. This phenomenon may have occurred because DG1 is a negatively charged polymer. Therefore, to obtain nanoparticles with high EE and LC, small particle size, and steady-state, the DG1 concentration of 7.2 mg/mL was selected to prepare DG1-NPs and DG1/Cur-NPs.

### 2.4. Effect of Cur Concentration

As shown in Table 6, nanoparticles size decreases with Cur concentration at Cur concentrations below 0.24 mg/mL and increases at Cur concentrations above 0.24 mg/mL. The zeta potential of nanoparticles increases with Cur concentration. In addition, The EE of Cur decreased with Cur concentration. The EE of the nanoparticles of Cur reached a maximum of 0.3% at a concentration of 0.24 mg/mL of Cur and decreased to 0.23% when the concentration of Cur increased to 0.48 or even 0.72 mg/mL. This may have occurred because the only form of the diketone group of Cur in solution is the enol, which leads to increased hydrophobicity and repels aqueous environments [30]. Therefore, the Cur concentration of 0.24 mg/mL was considered ideal for preparing DG1/Cur-NPs with high LC and small size.

### 2.5. Characterisation of Clam Heparinoid Nanoparticles

#### 2.5.1. SEM Analysis

**Figure 1:**
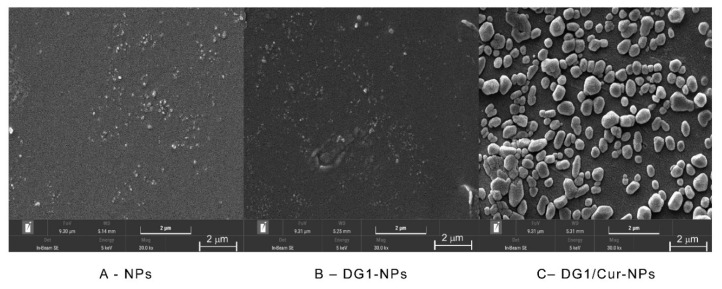
SEM analysis of NPs, DG1-NPs, and DG1/ Cur-NPs.

SEM images of NPs, DG1-NPs, and DG1/Cur-NPs are shown in Figure 1. NPs, DG1-NPs, and DG1/Cur-NPs were all spherical or sphere-like in shape. Compared with NPs and DG1-NPs, the particle size of DG1/Cur-NPs was distinctly bigger, and an agglomerated morphology was observed.

#### 2.5.2. FITR Analysis

**Figure 2:**
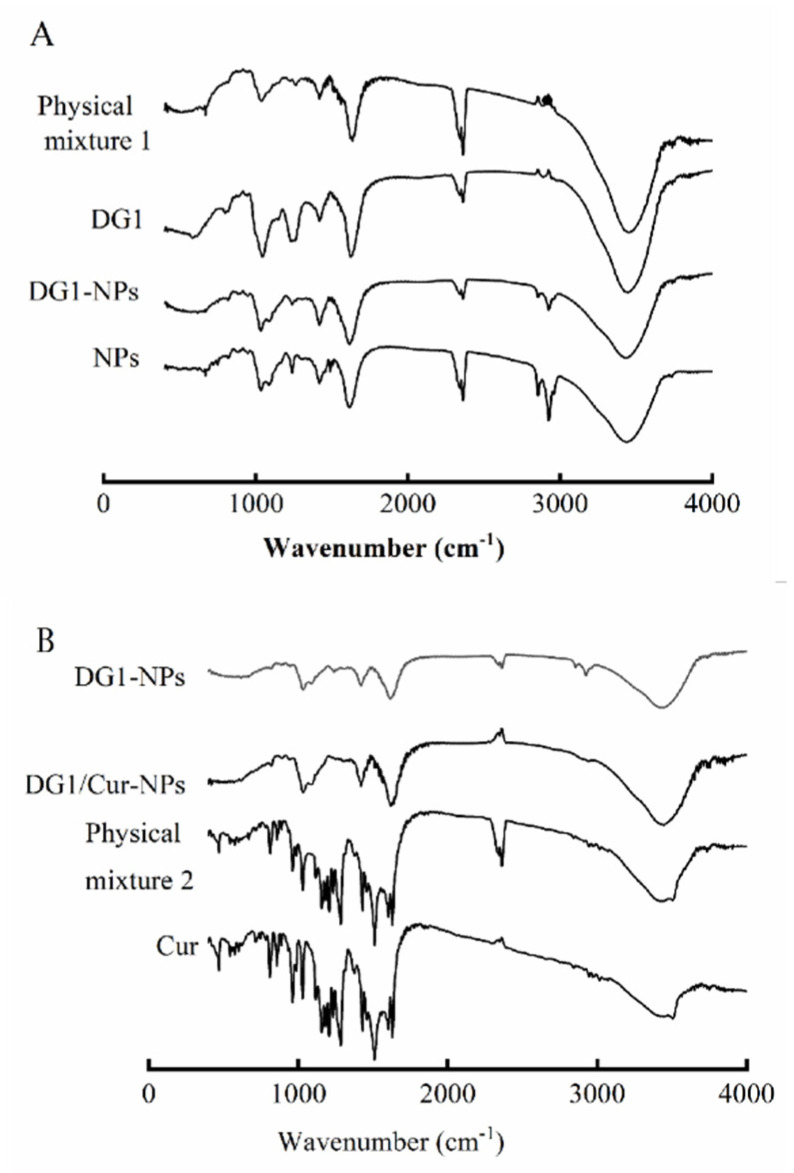
Infrared spectral analysis of DG1-NPs (**A**) and DG1/ Cur-NPs (**B**). Physical mixture 2: a formulated mixture of Cur and DG1-NPs; Physical mixture 1: a formulated mixture of DG1 and NPs.

FTIR was used to characterise the interactions between DG1 or Cur and polymers in the nanoparticles. As shown in Figure 2, the FTIR spectra of DG1 showed broadband at 3458 cm^−1^, which corresponded to O-H and N-H stretching. The band at 2987 cm^−1^ represented the C-H stretching of the aliphatic hydrocarbon side chain. The peak for the amide group C = O asymmetric stretching and N-H angle vibration occurred at 1627 cm^−1^. The band at 1427 cm^−1^ corresponded to the C-N stretching of the amide of the ether bond, whereas the peak at 1261 cm^−1^ was attributed to the S = O asymmetric stretching of the sulfonic acid group. The band at 1151 cm^−1^ corresponded to the C-O stretching of cyclopropyl ether. The band at 1051 cm^−1^ represented the C-O-C and C-OH stretching of the pyranose ring ether bond, whereas the peaks at 950 and 887 cm^−1^ are the characteristic peaks of heparin [8,31]. In the NPs spectra, the bands at 3458, 2987, 1627, 1427, 1261,1151, and 1051 cm^−1^ shifted slightly to 3439, 2964, 1625, 1427, 1234, 1093, and 1043 cm^−1^, respectively, and the respective maximum absorbing peaks at 3458, 1627, 1427, 1261, 1151, and 1051 cm^−1^ became lower in comparison to those in DG1. Physical mixture 1 is a mixture of clam heparin and NPs mixed in a dosage ratio. According to the FTIR spectra of Physical mixture 1 and DG1-NPs, the FTIR spectra of Physical mixture 1 is a superposition of DG1 and NPs peaks. The DG1-NPs and NPs spectra were similar. These results were consistent with those of Eleraky et al., which suggests that the micelle formation in the nanoparticles reduced some signals [32].

In the Cur spectra, the broadband at 3508 cm^−1^ was attributed to the O-H stretching of the hydroxyl group. The peaks at 1631 and 1510 cm^−1^ corresponded to the stretching and bending vibrations of C = C and C = O, respectively. The peaks at 1602, 1429, and 1286 cm^−1^ were attributed to the stretching vibration of the benzene ring skeleton, bending vibration of the alkene C-H plane, and stretching vibration of aromatic C-O, respectively. The peaks at 1163 and 808 cm^−1^ were associated with the aromatic C-H bending vibration, whereas the peak at 1029 cm^−1^ corresponded to the C-O-C stretching vibration [33]. After complexation with chitosan, alginate, and DG1, the bands at 3508, 1631, and 1429 cm^−1^ shifted slightly to 3448, 1624, and 1419 cm^−1^, respectively. The bands at 1602, 1286, 1510, 1163, 1029, and 808 cm^−1^ disappeared. Physical mixture 2 is a formulated mixture of Cur and DG1-NPS. After the addition of chitosan, alginate, and DG1, the peaks at 3508, 1631, 1602, 1510, 1429, 1286, 1163, 1029, and 808 cm^−1^ in the spectra of Cur alone remained in the spectra of Physical mixture 2. They shifted slightly to 3456, 1629, 1604, 1510, 1429, 1282, 1155, 1029, and 813 cm^−1^, respectively. These results suggest that the micelle formation in the nanoparticles led some signals to disappear, demonstrating the Cur entrapment in the DG1/Cur-NPs at the molecular level and implies that micelle formation during the nanoparticle preparation did not cause the breakdown of the Cur structure [27,34].

In summary, these findings demonstrate the entrapment of DG1 and Cur in the clam heparinoid nanoparticles at the molecular level.

#### 2.5.3. DSC Analysis

**Figure 3:**
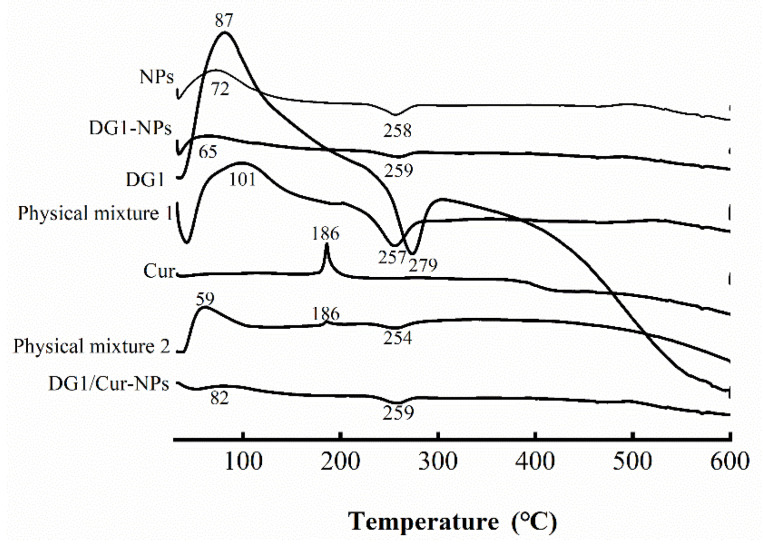
Differential scanning calorimetry (DSC) analysis of DG1-NPs and DG1/Cur-NPs. Physical mixture 2: a formulated mixture of Cur and DG1-NPs; Physical mixture 1: a formulated mixture of DG1 and NPs.

DSC is widely used for thermal analysis because when a new nanoparticle is formed, the melting and boiling points of the original sample are changed [35]. As shown in Figure 3, a sharp endothermic peak was observed at 87 °C, corresponding to the melting point of pure DG1, and an exothermic peak occurred at 279 °C. In NPs, there was an endothermic peak at 72 °C and an exothermic peak at 258 °C. DG1-NPs showed an endothermic peak at 65 °C and an exothermic peak at 259 °C. Physical mixture 1 showed an endothermic peak at 101 °C and an exothermic peak at 257 °C. For exothermic peaks, the peak values were in the following order: DG1 > Physical mixture 1 > NPs > DG1-NPs; whereas the exothermic peaks of DG1-NPs mixture, NPs, and DG1-NPs were all approximately 254 °C. The DSC thermogram of DG1 was more similar to that of Physical mixture 1 than that of DG1-NPS, which indicated the successful preparation of DG1-NPs.

In addition, for Cur, a sharp endothermic peak was observed at 186 °C, which is the melting point of pure Cur [36]. In Physical mixture 2, there were endothermic peaks at 59 °C and 186 °C and an exothermic peak at 254 °C. In contrast, no endothermic (melting) peak of Cur was observed in the DSC thermogram of DG1/Cur-NPs. The reduced intensity of the peak at 188 °C and the appearance of new peaks at 166 °C and 170 °C confirmed the reduced crystallinity of Cur and the formation of a new polymorph during the crystallisation procedure, as reported by Pantwalawalkar [37]. Sadeghi et al. reported that the melting peak of Cur disappeared in solid dispersion systems of Cur and PVP, which indicated that Cur was in an amorphous state in the precipitated samples [38]. Therefore, the absence of the endothermic melting peak of Cur suggested that Cur was encapsulated in the nanoparticles and was present in an amorphous form as a molecular dispersion or a disordered crystalline phase within the nanoparticle matrix.

### 2.6. Effect of DG1-NPs and DG1/Cur-NPs on Collagen plus Epinephrine-Induced Acute Pulmo-nary Thrombosis in Mice

Both epinephrine and collagen are potent platelet activators and cause significant platelet aggregation, and epinephrine has a significant vasoconstrictive effect [39]. A previous study reported that the injection of collagen and epinephrine caused the decrease in platelet count, aggregation of platelets in lung blood vessels, and death of mice owing to respiratory failure [40]. In addition, the thrombus produced in the mice lungs increased their lung mass, which increased their respective lung index.

The in vivo prevention of pulmonary thromboembolism in mice by clam heparinoid nanoparticles was investigated. As shown in Table 7, the protective effect of HP sodium and DG1 reached 40%, and that of DG1/Cur-NPs and DG1-NPs were both 50%, which is 1.25-fold higher than that of DG1 solution after oral administration. In addition, the lung coefficients of group M increased significantly (*p* < 0.01). In contrast, Lung index is the weight of the mouse lung as a percentage of the mouse’s body weight. The lung index of the DG1, DG1-NPs, and DG1/Cur-NPs groups for pulmonary embolism decreased significantly (*p* < 0.01). The lung coefficients of the DG1, DG1/Cur-NPs, and DG1-NPs groups were reduced sequentially. These results indicated that the capacity of clam heparinoid DG1 to protect mice against pulmonary embolism is comparable to that of heparin sodium at the same dose. That is, chitosan-alginate nanoparticles can improve the oral protective effect of clam heparinoid DG1 against collagen plus epinephrine-induced acute pulmonary thrombosis in mice. Moreover, NPs protected 10% of the mice from acute pulmonary embolism, and reduced lung index indicated that chitosan and SA have a slightly antithrombotic effect. Cur does not synergise with DG1 to exert antithrombotic effects, which may have occurred because the dose of Cur was low (approximately 0.12 mg/kg). Shi et al. also reported a thrombosis inhibition rate of 60.31% for Cur injected intraperitoneally at 50 mg/kg, comparable to that of aspirin gavaged at an equivalent dose (63.59%) in a rat carotid artery thrombosis model [41].

**Figure 4:**
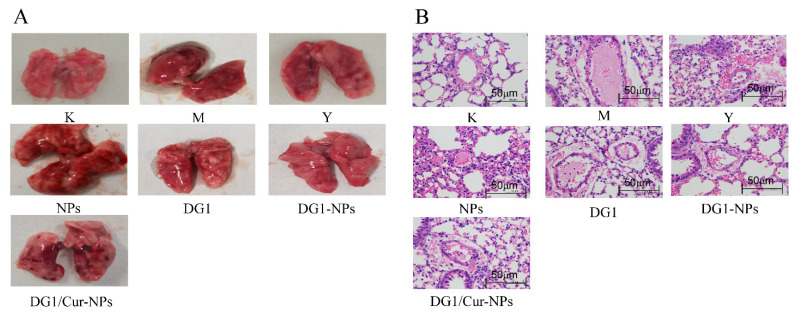
(**A**) Lung tissues of mice from each group, and respective (**B**) lung tissue sections (HE staining × 400). B group: the blank control group gavaged with water; M group: the model group gavaged with water and treated with collagen plus epinephrine; Y group: the positive control group gavaged with sodium heparin and treated with collagen plus epinephrine.

To further confirm the prevention of pulmonary thromboembolism by the clam heparinoid treatment, lung conditions were photographed, and cross-sections of mice lungs at similar locations were observed based on HE staining. Lungs from mice in group M showed many thrombi (Figure 4A). HE staining of lungs in group M showed thrombus in the capillaries, capillary congestion and oedema in the alveolar wall, and infiltration of lymphocytes and histiocytes, interspersed with varying amounts of inflammatory cells in the lungs of mice with pulmonary embolism (Figure 4B). Consistent with the protection effect and Iung index results, the treatment with DG1, DG1-NPs, DG1/Cur-NPs, sodium heparin, and NPs prevented these symptoms of acute pulmonary embolism from occurring in the lung of mice to different degrees (Figure 4A,B).

## 3. Discussion

Low molecular weight clam heparinoid DG1 with a molecular weight of 24.48 kDa are derived from vitamin catalytic free-radical depolymerisation of native clam heparinoid G2. We chose DG1 to prepare oral antithrombotic nanoparticles because of its strong oral antithrombotic activity and low molecular weight [9]. In this study, we analysed physicochemical properties that impact the preparation of DG1 nanoparticles. The results showed that DG1 is a water-soluble anionic polymer with a high content of sulphate groups and uronic acid and a high initial degradation temperature.

To prepare nanoparticles with small particle size, suitable potential, and high encapsulation rate and LC, we performed single-factor optimisation experiments on the formulation parameters of nanoparticles. The results showed that 0.6 mg/mL SA, 0.33 mg/mL CaCl2, 0.6 mg/mL CTS, 7.2 mg/mL DG1, and 0.24 mg/mL Cur were optimal formulation parameters for the preparation of clam heparinoid nanoparticles. Under these conditions, we prepared empty nanoparticles, NPs, DG1 only nanoparticles, DG1-NPs, and DG1 and Cur compliant nanoparticles, DG1/Cur-NPs. NPs presented a particle size of 191.8 nm. In comparison, Azevedo et al. [42] reported a mean size of approximately 168 nm for nanoparticles using similar methodologies and materials. This may be related to each solution component’s pH and final concentration mass ratio used to prepare these two nanoparticles. DG1-NPs presented particle size, zeta potential, PDI, LC, and EE of 210.2 ± 0.7 nm, −35.2 ± 1.5 mV, 0.66 ± 0.07, 94.52 ± 1.24%, and 13.90 ± 0.17 U/mg, respectively. Compared with the alginate/chitosan nanoparticles of vitamin B2 and chitosan/alginate nanoparticles containing curcumin diethyl disuccinate, DG1-NPs presented higher EE and more negative zeta potential, likely attributable to the water solubility and negative charge of DG1 [26,42]. The DG1/Cur-NPs presented particle size, zeta potential, and PDI of 339.7 ± 11.2 nm, −28.5 ± 2.0 mV, and 0.51 ± 0.04. The LC and EE of DG1 were 86.98 ± 1.13% and 13.10 ± 0.17 U/mg, whereas those of Cur were 21.04 ± 2.61% and 0.30 ± 0.04%, respectively. Compared with the curcumin diethyl disuccinate-only nanoparticles [27], the DG1/Cur-NP size was smaller and presented a lower Cur embedding rate and LC. In addition, compared with DG1-NPs, the particle size of DG1 was significantly larger, whereas its embedding rate and drug LC were lower. Therefore, it is necessary to further increase the DG1 and Cur LC of DG1/Cur-NPS by adjusting the pH of the solution, for instance, using surfactants.

In addition, we have characterised the nanoparticles. The results of SEM, FITR, and DSC indicated the entrapment of DG1 and Cur in the clam heparinoid nanoparticles at the molecular level, and Cur was loaded in the amorphous phase within the nanoparticle matrix. These results suggested that Cur is a hydrophobic compound, which is not highly soluble under neutral, acidic conditions [31,43]. However, it can be encapsulated in chitosan/alginate nanoparticles.

One of the characteristics of pulmonary embolism is “three highs”, i.e., high morbidity, high misdiagnosis, and high mortality [3]. The mice pulmonary embolism model can simulate pulmonary embolism well and has the characteristics of easy modeling and significant effect. As a classical antithrombotic, aspirin showed a protective effect by 50–80% at a dose of 20 mg/kg and above [44,45,46]. Tang et al. discovered that the protective effect of enoxaparin-loaded LPHN2 was 50.0%, 2.99-fold higher than that of enoxaparin solution (16.70%) after oral administration, indicated that lipid–polymer hybrid nanoparticles are effective in improving oral absorption and the inhibition effect of enoxaparin against thrombin-induced thrombosis [14]. Furthermore, the Viscera index can be used to detect pathological changes in animals [47]. Shen et al. discovered that aspirin eugenol ester significantly reduces lung coefficients in mice with pulmonary embolism (0.98 ± 0.07% vs. 0.84 ± 0.04%) [48]. In this study, we observed that the protective effect of DG1/Cur-NPs and DG1-NPs were both 50%, which is 1.25-fold higher than that of DG1 solution. The lung coefficients of pulmonary embolism mice in the DG1/Cur-NPs and DG1-NPs groups were slightly lower than those in the DG1 group (0.77 ± 0.15% and 0.79 ± 0.18% vs. 0.80 ± 0.26%). Thus, our results suggest that the chitosan/alginate nanoparticles enhanced the oral antithrombotic activity of DG1. However, Cur did not further enhance this effect under the conditions of this study. Nonetheless, studying their oral antithrombotic mechanisms at the molecular and genetic levels is important. The oral antithrombotic activity of nanoparticles can also be further evaluated by mice carrageenin-induced thrombosis model, rat carotid thrombosis model, rat venous thrombosis model, and other animal models.

Chitosan/alginate nanoparticles by ionotropic pre-gelation are characterised by a compact chitosan film overlying the calcium-alginate pre-gel, enabling controlled release and strong absorption of nanoparticles in the intestine [49,50]. However, the in vitro simulated digestion, Cao2 cell model, and other means were not used in this study to investigate the basic characterisations in terms of release of nanoparticles, biocompatibility, and disponibility of DG1 or Cur after oral administration, which is the next step we need to investigate.

## 4. Materials and Methods

### 4.1. Materials

Clam heparinoid DG1 was prepared as described by Chen et al. [51]. Chitosan (91.5% degree of deacetylation), alginic acid sodium salt (analytical research (AR) grade, 98% degree of purity), Cur (Biological Grade (BR), 98% degree of purity), and collagen (isolation from cow heel, and molecular weight greater than 300,000 g/mol) were purchased from Shanghai Yuanye Biological Co., Ltd. (Shanghai, China). AR grade acetic acid, hydrochloric acid, sodium hydroxide, and calcium chloride were obtained from Xilong Science Co., Ltd. (Guangzhou, China). Epinephrine was purchased from Sanma Veterinary Medicine Co., Ltd. (Harbin, China).

Male Kunming mice (20–22 g) were provided by Zhuhai Bestest Biotech Co., Ltd. (Zhuhai, China). The animals were housed under a 12 h light-dark cycle at a constant ambient temperature of 22−25 °C and humidity of 55 ± 5%, with normal chow and water ad libitum. They were allowed to acclimatise for one week before the experiments began. All animal care and experimental protocols were strictly followed according to the guidelines of the Laboratory Animal Center of Guangdong Ocean University and were approved by the Institutional Animal Care and Use Committee of Guangdong Ocean University (No. GDOU-LAE-2021-003).

### 4.2. Determination of Physicochemical Properties of DG1

The uronic acid content of DG1 was determined by a modified sulphate-carbazole method [52]. The sulphate ester content of DG1 was estimated according to the Gel-BaCl_2_ colorimetric method [53].

A solution of clam heparinoid DG1 at a 0.01 mg/mL concentration was prepared and filtered through a 0.2 μm membrane. The particle size and zeta potential of DG1 were determined using a Zetasizer model Nano-ZS (Nano-ZS90, Malvern Instruments, England).

Thermal stability analysis of DG1 was carried out using a simultaneous thermal analyser (TA Q2000, TA Instruments, USA). The samples were analysed for thermal weight loss (TG) in the temperature range 20–600 °C under N_2_ atmosphere at a 50 mL/min flow rate and a ramp rate of 10 °C/min.

### 4.3. Preparation and Optimisation of Clam Heparinoid Nanoparticles

Clam heparinoid nanoparticles were prepared by the ionic cross-linking method, as reported by Bhunchu et al. [27]. For that, the clam heparinoid DG1 solution (1 mL) and Cur ethanol solution (1 mL) were added dropwise to the SA solution (20 mL) using a peristaltic pump (HN-2, Shanghai Huxi Analytical Instrument Factory Co. Ltd., Shanghai, China) at an addition rate of 5 rpm under mechanical stirring at 1000 rpm for 10 min, followed by dropwise addition of calcium chloride solution (4 mL) and continued stirring for 10 min. After sonication at a frequency of 40 kHz and sonic power of 20 W (JP-040S, Shenzhen Clean Union Cleaning Equipment Co. Ltd., Shenzhen, China) for 5 min, 4 mL of CTS acetic acid solution (1%, *v/v*) at varying concentrations were added dropwise to the resultant calcium-alginate pre-gel with continuous stirring at 1000 rpm for 10 min, followed by standing for 20 min, centrifugation at 3000 rpm for 10 min to remove the insoluble material, and removal of the supernatant, which was freeze-dried to produce the DG1/Cur-NPs. The DG1-NPs were prepared by omitting the Cur solution, and NPs were prepared by omitting the Cur and DG1 solutions.

The NPS characteristics were examined in formulations using various concentrations of SA, calcium chloride, and CTS. Subsequently, the characteristics of DG1-NPs and DG1/Cur-NPs were examined in formulations using various concentrations of DG1 and Cur, respectively. We investigated SA concentration of 0.2, 0.4, 0.6, 0.8, 1.2, and 1.6 mg/mL; calcium chloride concentrations of 0.033, 0.066, 0.165, 0.33, 0.66, 1.32, 1.98, and 2.64 mg/mL; CTS concentrations of 0.15, 0.3, 0.45, 0.6, 0.75, 0.9, 1.05, and 1.2 mg/mL; DG1 concentrations of 2.4, 4.8, 7.2, 9.6, 12, and 24 mg/mL; and Cur concentrations of 0.03, 0.06, 0.12, 0.24, 0.48, 0.96, and 1.2 mg/mL. When one parameter was varied, the others remained constant. The selection of the optimum formulation was based on the particle size, size distribution (PDI), zeta potential, encapsulation efficiency, and loading capacity of the nanoparticles.

### 4.4. Characterisation of Clam Heparinoid Nanoparticles

The particle size and size distribution based on the PDI and zeta potential were determined using a Zetasizer Nano-ZS model (Nano-ZS90, Malvern Instruments, England). Subsequently, a small number of nanoparticles were placed on an aluminium foil sheet, dried, and sprayed with gold treatment. The morphologies of NPs, DG1-NPs, and DG1/Cur-NPs were then visualised by SEM (EM-7610-F, Japan Electronics Corporation, Japan).

A 20 mL aqueous solution containing 10 mg of lyophilised nanoparticles was placed in a 25 mL volumetric flask. After the pH of the supernatant was adjusted to 6.5 with 2 mol/L HCl solution (using distilled water), the supernatant was sonicated at 500 W and 100 Hz for 10 min. Subsequently, 6 mL of this solution was placed in a 10 mL volumetric flask, which was then filled with distilled water, and the potency of the clam heparinoid DG1 in DG1/Cur-NPs and DG1-NPs was determined by the azure A method [54]. For that, 1 mL of this solution was mixed with 4 mL of ethanol, and the amount of Cur in DG1/Cur-NPs was assayed as described by Ma et al. [55] (with modifications). The encapsulation efficiency was calculated from the amount of DG1 or Cur in the nanoparticles as a percentage of the total amount of DG1 or Cur initially added to the formulation. The loading capacity was calculated from the amount of DG1 or Cur in the nanoparticles as a percentage of the total dry mass. The equations were as follows:

Encapsulation efficiency (%) = DG1 or Cur in solution × 100%/DG1 or Cur initial(1)

Loading capacity = DG1 or Cur in solution × 100%/total dry mass of nanoparticles(2)

The interaction and compatibility of DG1 or Cur with other components in the DG1-NPs and DG1/Cur-NPs formulation, respectively, were analysed based on the FTIR spectra measured on a Bruker Tensor 27 FTIR spectrometer using the OPUS 7.5 software. The samples were dried under an infrared lamp for 2 h, mixed with KBr, grounded, and pressed into 1 mm pellets for FTIR spectral analysis at 4000–400 cm^−1^.

The physical state of DG1 or Cur in the nanoparticles was analysed by a simultaneous thermal analyser (TA Q2000, TA Instruments, USA). An inert atmosphere was maintained by purging nitrogen at a 20 mL/min flow rate. The instrument conditions followed the method described by Murthy et al. with modifications [56]. An appropriate amount of lyophilised nanoparticles was sealed in an aluminium pan and scanned at 30–600 °C, at a heating rate of 10 °C/min.

### 4.5. Effect of DG1-NPs and DG1/Cur-NPs on Collagen Plus Epinephrine-induced Acute Pulmonary Thrombosis in Mice

The antithrombotic activity of DG1-NPs and DG1/Cur-NPs was evaluated by establishing an acute pulmonary embolism model in mice, as reported by Choi et al. [46]. The experimental mice were randomly divided into seven groups (*n* = 10), namely blank (B); sodium heparin (Y); model (M); DG1; NPs; DG1-NPs; and DG1/Cur-NPs groups. Except for group B, the other groups were gavaged with NPS, sodium heparin, DG1, DG1-NPs, and DG1/Cur-NPs at a single dose of 5560 U/k. Two hours after administration, all groups were intravenously injected with 10 mL/kg of a mixture of collagen (1 mg/mL) and epinephrine (0.1 mg/mL) via the tail vein to induce hind limb paralysis or death. The number of dead or paralysed mice was recorded within 20 min. The results show a percentage of protection. All surviving mice were sacrificed immediately after the experiment. All mice were dissected. Their lung tissues were collected, washed several times with saline, placed on clean filter paper to absorb the water, weighed, photographed, and then stained in HE sections, as reported by Gupta et al. [44] and Men et al. [57].

The equation of Lung index was Lung index = (Wl/Wb) × 100%, where Wl is the weight of the mouse lung and Wb is the weight of the mouse.

### 4.6. Statistical Analysis

The results are expressed as mean ± standard deviation. The experimental data were subjected to analysis of variance for a completely random design, and three samples were prepared for each assay. Data processing was performed using SPSS 17 and Origin 8.

## 5. Conclusions

Our results demonstrate that the molecular weight of heparin impacts its antithrombotic activity. Furthermore, molecular weight is an essential chemical characteristic for clam heparinoids administered by the oral routine. Further, DG1 had better absorption and antithrombotic activities than G2. Considering the large molecular weight of G2, the medium-molecular-weight clam heparinoid DG1 seems to be an attractive choice as an oral antithrombotic agent to prevent thrombotic disease.

## Figures and Tables

**Table 1:** Physicochemical properties of DG1.

Name of Sample	Content of Sulfate Radical (%)	Content of Uronic Acid (%)	Zeta Potential (mV)	Particle Size (nm)	Maximum Initial Degradation Temperature (°C)
DG1	24.74 ± 0.64	22.38 ± 0.93	−25.1 ± 1.0	183.9 ± 6.2	246

**Table 2:** Effects of SA concentration on the characteristics of nanoparticles with a fixed mass concentration ratio of sodium alginate to CaCl_2_ to chitosan of 1:0.22:0.15 (*n* = 5).

SA Concentration (mg/mL)	Particles Size (nm)	PDI ^a^	Zeta Potential (mV)
0.2	180.0 ± 2.7	0.23 ± 0.01	20.5 ± 4.8
0.4	186.0 ± 2.7	0.40 ± 0.06	−9.5 ± 0.3
0.6	236.5 ± 6.6	0.52 ± 0.03	−26.4 ± 1.1
0.8	261.9 ± 46.2	0.71 ± 0.11	−34.1 ± 1.3
1.0	293.6 ± 14.9	0.83 ± 0.17	−37.2 ± 1.3

^a^ PDI: polydispersity index.

**Table 3:** Effects of calcium chloride concentration on nanoparticles on the characteristics of nanoparticles with a fixed mass concentration ratio of sodium alginate to CaCl_2_ to chitosan of 1:0.22:0.15, a SA concentration of 0.6 mg/mL (*n* = 5).

CaCl_2_ Concentration (mg/mL)	Particles Size (nm)	PDI ^a^	Zeta Potential (mV)
0.06	185.5 ± 4.1	0.57 ± 0.04	−38.7 ± 1.3
0.165	184.2 ± 4.0	0.49 ± 0.03	−32.2 ± 1.4
0.33	171.9 ± 1.1	0.50 ± 0.00	−27.9 ± 1.9
0.66	246.2 ± 4.0	0.60 ± 0.07	−27.2 ± 0.1
1.32	352.8 ± 5.8	0.40 ± 0.01	−20.9 ± 0.9

^a^ PDI: polydispersity index.

**Table 4:** Effects of chitosan concentration on nanoparticles on the characteristics of nanoparticles with a fixed mass concentration ratio of sodium alginate to CaCl_2_ to chitosan of 1:0.22:0.15, a SA concentration of 0.6 mg/mL, and a CaCl_2_ concentration of 0.33 mg/mL (*n* = 5).

CTS Concentration (mg/mL)	Particles Size (nm)	PDI ^a^	Zeta Potential (mV)
0.15	147.3 ± 3.8	0.60 ± 0.04	−32.3 ± 0.3
0.3	181.8 ± 4.1	0.54 ± 0.04	−30.7 ± 1.5
0.45	196.8 ± 4.0	0.59 ± 0.01	−30.6 ± 0.5
0.6	191.8 ± 3.2	0.34 ± 0.00	−29.9 ± 0.5
0.75	175.9 ± 3.3	0.48 ± 0.03	−29.9 ± 0.4

^a^ PDI: polydispersity index.

**Table 5:** Effects of clam heparin DG1 concentration on DG1-NPs with a fixed mass concentration ratio of sodium alginate to CaCl_2_ to chitosan of 1:0.22:0.15, a SA concentration of 0.6 mg/mL, a CaCl_2_ concentration of 0.33 mg/mL, and a CTS concentration of 0.6 mg/mL (*n* = 5).

DG1 Concentration (mg/mL)	Particles Size (nm)	PDI ^a^	Zeta Potential (mV)	Encapsulation Efficiency (%)	Loading Capacity (U/mg)
2.4	210.2 ± 0.7	0.47 ± 0.01	−31.8 ± 1.1	91.37 ± 0.00	6.06 ± 0.00
4.8	248.3 ± 8.5	0.50 ± 0.01	−31.2 ± 0.8	92.37 ± 1.62	10.51 ± 0.11
7.2	252.4 ± 0.9	0.49 ± 0.03	−33.8 ± 1.0	94.52 ± 1.24	13.90 ± 0.17
9.6	366.6 ± 21.1	0.66 ± 0.07	−35.2 ± 1.5	98.09 ± 0.47	18.01 ± 0.08
12	225.5 ± 4.4	0.51 ± 0.03	−35.2 ± 1.6	90.09 ± 0.37	18.89 ± 0.07

^a^ PDI: polydispersity index.

**Table 6:** Effects of curcumin concentration on DG1/Cur-NPs with a fixed mass concentration ratio of sodium alginate to CaCl_2_ to chitosan of 1:0.22:0.15, a SA concentration of 0.6 mg/mL, a CaCl_2_ concentration of 0.33 mg/mL, and a CTS concentration of 0.6 mg/mL, and a DG1 concentration of 7.2 mg/mL (*n* = 5).

Cur Concentration (mg/mL)	Particles Size (nm)	PDI ^a^	Zeta Potential (mV)	Cur Encapsulation Efficiency (%)	Cur Loading Capacity (%)
0.06	465.3 ± 32.7	0.59 ± 0.04	−33.7 ± 0.3	27.90 ± 3.33	0.10 ± 0.01
0.12	354.9 ± 33.4	0.62 ± 0.06	−28.5 ± 2.0	23.81 ± 1.91	0.17 ± 0.01
0.24	339.7 ± 11.2	0.51 ± 0.04	−28.4 ± 1.3	21.04 ± 2.61	0.30 ± 0.04
0.48	1840.3 ± 223.4	1.00 ± 0.00	−27.9 ± 0.6	8.24 ± 0.38	0.23 ± 0.01
0.72	2079.0 ± 162.8	1.00 ± 0.00	27.1 ± 0.6	5.57 ± 0.77	0.23 ± 0.03

^a^ PDI: polydispersity index.

**Table 7:** Effects of oral administration of different heparin nanoparticles on pulmonary thromboembolism and pulmonary coefficient (*n* = 10).

Group	Protection Effect (%)	Iung Index
K ^b^	-	0.62 ± 0.08
M ^b^	0	1.06 ± 0.30 ## ^a^
Y ^b^	40	0.91 ± 0.22
NPs	10	0.95 ± 0.24
DG1	40	0.80 ± 0.26 ** ^a^
DG1-NPs	50	0.77 ± 0.15 ** ^a^
DG1/Cur-NPs	50	0.79 ± 0.18 ** ^a^

^a^ Statistical significance: ** *p* < 0.01, as compared with model (M) group. ## *p* < 0.01, as compared with blank control (K) group. ^b^ B group: the blank control group gavaged with water; M group: the model group gavaged with water and treated with collagen plus epinephrine; Y group: the positive control group gavaged with sodium heparin and treated with collagen plus epinephrine.

## Data Availability

The data presented in this study are available on request from the corresponding author. The data are not publicly available due to public availability violating the consent given by research participants.
